# Causal associations between gut microbiota and Cholestatic liver diseases: a Mendelian randomization study

**DOI:** 10.3389/fmed.2024.1342119

**Published:** 2024-01-24

**Authors:** Jiaqi Yang, Gang Ma, Kemei Wang, Hui Yang, Shuangshuang Jiang, Qingling Fan, Xinmin Zhou, Guanya Guo, Ying Han

**Affiliations:** Department of Digestive Diseases, Xijing Hospital, Fourth Military Medical University, Xi’an, China

**Keywords:** primary sclerosing cholangitis, primary biliary cholangitis, Mendelian randomization, cholestatic liver diseases, gut microbiota

## Abstract

**Background:**

The etiological factors of Cholestatic Liver Diseases especially primary sclerosing cholangitis (PSC) and primary biliary cholangitis (PBC) are not fully illustrated. It has been reported in previous observational studies that gut microbiota are associated with cholestatic liver diseases. However, there is uncertainty regarding the causality of this association. By using Mendelian randomization, this study aimed to examine the causal impact of gut microbiota on cholestatic liver diseases.

**Methods:**

From large-scale genome-wide association studies, genetic instruments for each gut microbiota taxa as well as primary biliary cholangitis and primary sclerosing cholangitis were developed. Subsequently, we conducted a two-sample Mendelian randomization analysis, supplemented by multiple *post hoc* sensitivity analyses. Additionally, we performed reverse MR analyses to investigate the possibility of the reverse causal association.

**Result:**

This two-sample MR study indicated that the *order Bacillales, family Peptostreptococcaceae, family Ruminococcaceae, genus Anaerotruncu* was associated with a decreased risk of developing PBC, and that *order Selenomonadales, family Bifidobacteriaceae* may be factors that increase the risk of PBC. On the other hand, we also identified *order Selenomonadales, family Rhodospirillaceae, and genus RuminococcaceaeUCG013* were positively associated with PSC. The *order Actinomycetales, family Actinomycetaceae, genus Actinomyces, genus Alloprevotella, genus Barnesiella*, and *genus Peptococcus* were found negative associations with the risk of PSC. The reverse MR analysis demonstrated no statistically significant relationship between PBC, PSC and these specific gut microbial taxa.

**Conclusion:**

Our findings offered novel evidence that the abundance of particular bacteria contributes to the risk of PBC and PSC, which may contribute to more effective approaches to PBC and PSC therapy and prevention.

## 1 Introduction

Cholestatic liver disease (CLD) refers to a group of disorders in which bile synthesis, secretion, and excretion are compromised for a variety of reasons (1). CLD dominantly includes primary sclerosing cholangitis (PSC) and primary biliary cholangitis (PBC). PBC, a chronic cholestatic liver disease, is hallmarked by non-suppurative inflammation within the small intrahepatic bile duct (2). PSC, a rare cholestatic liver disease, could result in bile duct fibrosis and strictures. In contrast to PBC, which has a female predominance, the majority of PSC patients are male (3). Up to 80% of PSC patients also suffer from IBD, indicating the involvement of the gut-liver axis in PSC (4). Patients with PBC can effectively control the disease by taking medications such as Ursodeoxycholic Acid, Bezafibrate and Fenofibrate (5–7). However, there is currently no satisfactory treatment for PSC.

Though the exact mechanism underlying PBC and PSC is still not fully illustrated, it is reported that genetics, environment, immune factors, gut microbiota, and individual susceptibility may all contribute to the development of these diseases (4, 8, 9).

Environmental factors are thought to cause PBC and PSC in individuals who are genetically predisposed, resulting in a loss of tolerance to self-antigens. Molecules derived from microbiota can activate the immune system and lead to autoimmune inflammation (10). According to recent research, gut microbiota dysbiosis can affect the immune system leading to autoimmune diseases such as celiac disease, inflammatory bowel disease, and cholestatic liver diseases (11–13). In numerous studies focused on the gut-liver axis, a link has been established between gut microbiota dysbiosis and PBC and PSC pathophysiology (14–16). Research has indicated a significant reduction in the abundance of microbiota in individuals with PBC and PSC compared to healthy controls (17, 18). However, some bacterial genera, such as *Haemophilus*, *Veillonella*, *Clostridium*, and *Bifidobacterium*, are increased in PBC patients compared to healthy controls (19, 20), while *Lactobacillus*, *Streptococcus*, and *Veillonella* proportions are higher in PSC patients than healthy controls (21).

Nevertheless, it is important to note that observational studies are often influenced by confounding factors. Moreover, the aforementioned studies have examined different populations with varying dietary habits. Therefore, these cross-sectional studies do not allow for definitive conclusions to be drawn.

Mendelian randomization (MR) is an innovative approach to investigate the association between an exposure and a noteworthy outcome (22). Alleles are randomly allocated, according to Mendelian’s laws of inheritance, and genotypes are fixed at conception. Thus, confounders and reverse causality are unlikely to affect the causal relationship. MR analysis exploited common genetic variations to represent a modifiable environmental exposure, which has become widely used to investigate potential causal relationships between environmental exposures and outcomes. In two-sample MR analysis, single-nucleotide polymorphisms (SNPs) can serve as instrumental variables (IVs) to investigate casual associations between exposures and outcomes (23).

In summary, the causality of the associations between the gut microbiota and PBC and PSC remains inconclusive. In this study, we conducted a two-sample Mendelian randomization analysis using comprehensive summary statistics from large-scale genome-wide association studies (GWAS) of gut microbiota (GM), PBC and PSC to conduct this question.

## 2 Materials and methods

### 2.1 Study design

**Figure 1:**
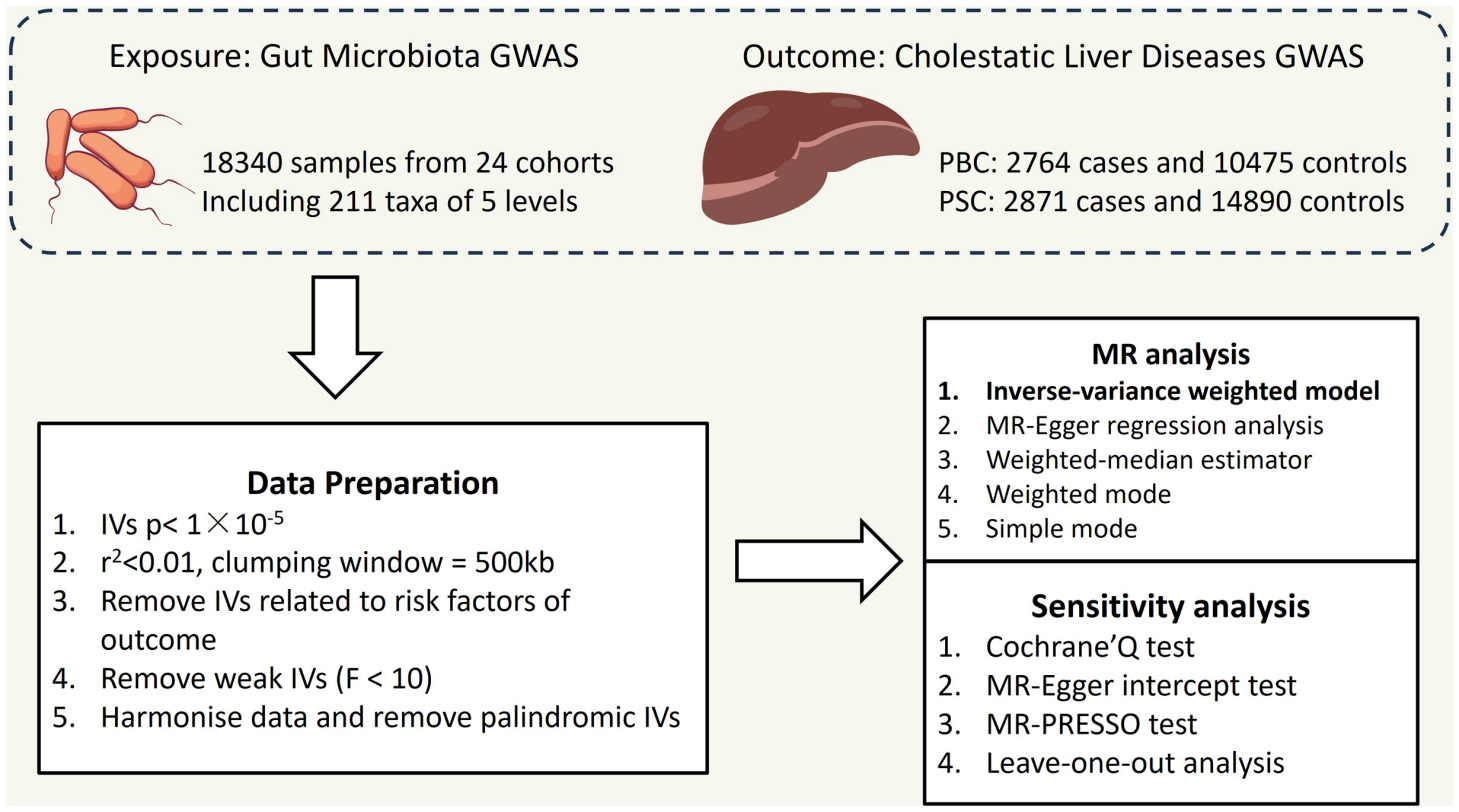
Overall flow chart of this study PBC: primary biliary cholangitis; PSC: primary sclerosing cholangitis; IVs: instrumental variables.

As shown in Figure 1, we implemented a two-sample MR to explore casual associations between CLD (specifically PBC and PSC) and gut microbiota.

### 2.2 Data source for exposure

MiBioGen consortium was formed to study the role of human genes in gut microbiota composition. Our study used the latest gut microbiota GWAS data extracted from 18,340 individuals. In this GWAS study, genetic variants associated with 211 GM taxa (9 phyla, 16 classes, 35 families, and 131 genera) were identified. Here is a link to download GWAS summary statistics for GMs.^1^

### 2.3 Data source for the outcomes

In two European cohorts, we obtained GWAS summary statistics for PBC and PSC. The PBC GWAS dataset comprises 10,475 controls and 2,764 cases (24), and the PSC GWAS dataset contains 2,871 cases and 14,890 controls (25).

### 2.4 Identification of IVs

To confirm the causal association of PBC and PSC with the gut microbiota, suitable IVs were chosen by implementing the subsequent quality control measures.

Firstly, we selected the IVs that are strongly correlated with GM taxa. As the initial threshold (*p* < 5 × 10^−8^) did not yield a sufficient number of IVS, we opted for a relatively lenient threshold (*p* < 1 × 10^−5^) to ensure enough IVs for obtaining robust results. Additionally, linkage disequilibrium (LD) correlation coefficient was set to *r*^2^ < 0. 01 and clumping window >500 kb to mitigate LD. Then, palindromic SNPs were removed from the IVs. Lastly, to evaluate weak instrumental bias, we calculated the *F* statistic of IVs. An F-statistic greater than 10 in MR analyses indicated no weak instrumental bias.

### 2.5 Statistical methods

Defined as the primary MR method for inferring causality, the inverse variance weighted (IVW) method is an extension of the Wald ratio method (26). In addition to IVW, we also applied four other MR methods: simple mode, weighted median, MR-Egger, and weighted mode. MR Egger’s method could also be used to detect directional pleiotropy (27).

We also conducted several sensitivity analyses to validate the stability of the causal association. We first performed Cochrane’s *Q* test to evaluate the heterogeneity across all selected SNPs. Additionally, we used MR-PRESSO and the MR-Egger intercept test for detection purposes. To assess the robustness of our results, we performed a leave-one-out analysis. All *p* < 0.05 was thought to be significant. Reverse MR analysis was employed to confirm the causal direction. It followed similar methods as forward MR, but PBC, PSC was regarded as the exposures, and we extracted SNPs associated with PBC, PSC as the IVs (*p* < 5 × 10–8).

We performed all analyses in this study using R software (version 4.2.1). We utilized R packages including the “ggplot2,” “TwoSampleMR,” and “MRPRESSO” for our MR study.

## 3 Results

### 3.1 Genetic IVs for gut microbiome

There were 2,934 SNPs as IVs linked to 211 GM taxa (9 phyla, 16 classes, 35 families, and 131 genera) in our MR study. The *F*-values of the selected SNPs ranged from 14.59 to 88.43, indicating a lower risk of weak instrument bias. The detailed information of all SNPs is shown in Supplementary Table 1.

### 3.2 PBC

Seven bacterial taxa were identified to be associated with PBC. It was determined that two of these taxa may increase the risk of PBC, specifically containing the *order Selenomonadales* (IVW OR = 2.13, 95% CI 1.10–4.14, *p* = 0.026), *family Bifidobacteriaceae* (IVW OR = 1.40, 95% CI 1.06–1.85, *p* = 0.019).

**Figure 2:**
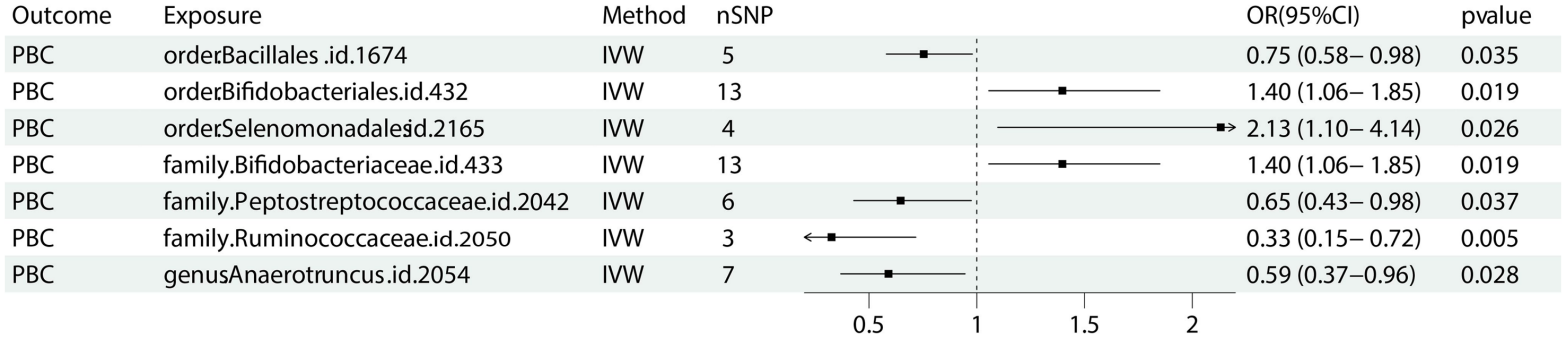
Forest plot of GM taxa associated with PBC identified by IVW method. PBC: primary biliary cholangitis; nSNP, number of the single nucleotide polymorphisms; IVW: inverse variance weighted method.

On the contrary, 4 taxa including *order Bacillales* (IVW OR = 0.75, 95%CI 0.58–0.95, *p* = 0.035), *family Peptostreptococcaceae* (IVW OR = 0.65, 95% CI 0.43–0.98, *p* = 0.037), *family Ruminococcaceae* (IVW OR 0.33, 95% CI 0.15–0.72, *p* = 0.005) and *genus Anaerotruncu* (IVW OR 0.59, 95% CI 0.37–0.95, *p* = 0.28) are identified as having negative associations with PBC, and may causally reduce the risk of PBC (Figure 2). Other results are shown in Supplementary Table 2.

**Figure 3:**
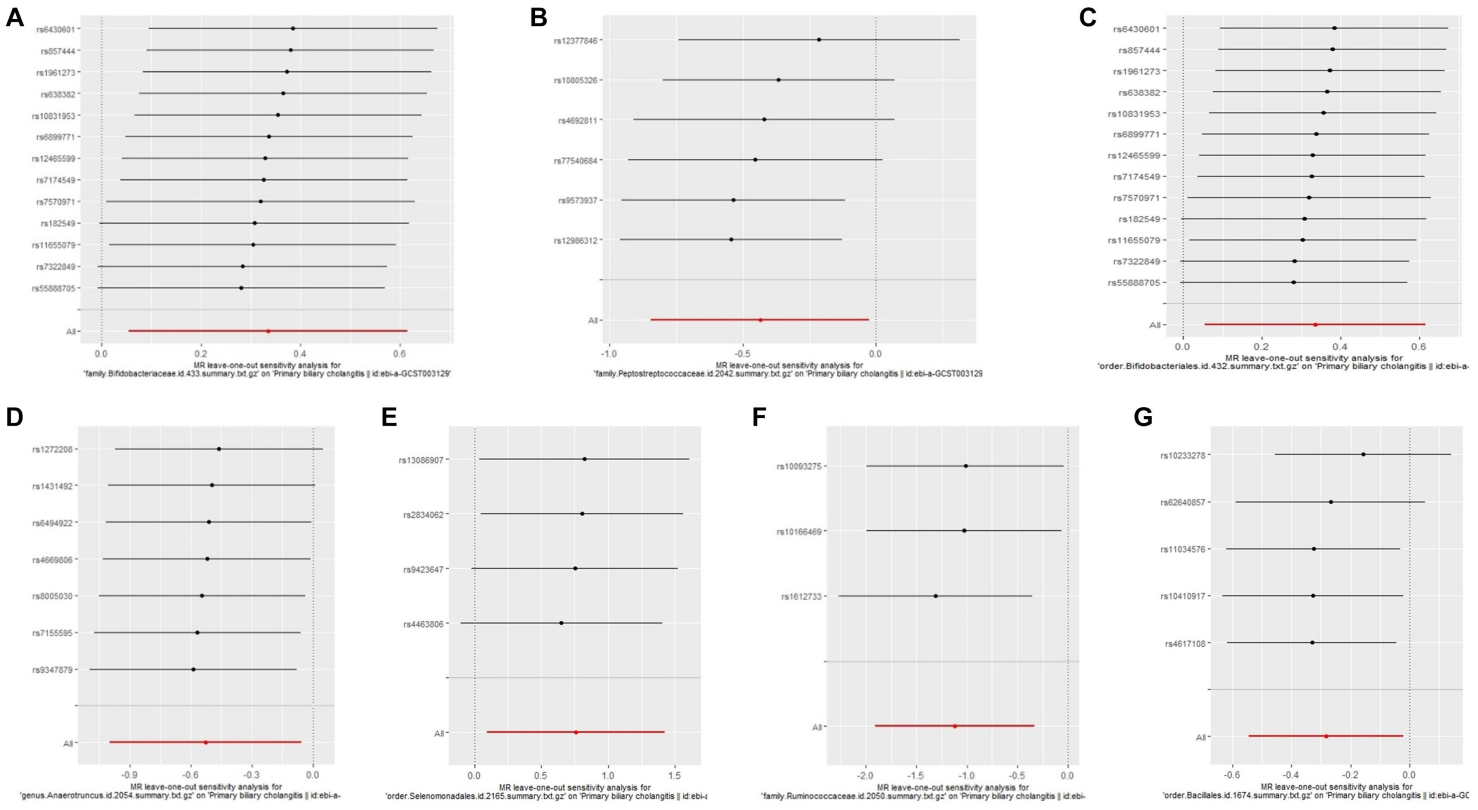
Leave one out analysis of the MR results of GM taxa associated with PBC. **(A)** family. Bifidobacteriaceae.id.433, **(B)** family.Peptostreptococcaceae.id.2042, **(C)** order.Bifidobacteriales.id.432, **(D)** genus.Anaerotruncus.id.2054, **(E)** order.Selenomonadales.id.2165, **(F)** family.Ruminococcaceae.id.2050, **(G)** order.Bacillales.id.1674.

The Cochrane’s *Q* test, the MR-Egger intercept test, and the MR-PRESSO test did not indicate any obvious heterogeneity in selected SNPs (Table 1) and showed that there is no pleiotropy or outliers (*p* > 0.05). Examination of forest plots and scatter plots was conducted (Supplementary Figures 1, 2). Finally, the leave-one-out method confirms our main results’ robustness (Figure 3).

**Table 1:** Sensitivity analysis of gut microbiota on PBC.

Outcome	Exposure	Cochrane’s Q	*p*	Egger_intercept	*p*	RSSobs	*p*
PBC	order.Bacillales.id.1674	3.585	0.465	−0.082	0.333	5.799	0.516
PBC	order.Bifidobacteriales.id.432	10.151	0.603	−0.016	0.654	11.500	0.656
PBC	order.Selenomonadales.id.2165	0.376	0.945	0.011	0.955	0.648	0.957
PBC	family.Bifidobacteriaceae.id.433	10.151	0.603	−0.016	0.654	11.548	0.636
PBC	family.Peptostreptococcaceae.id.2042	5.458	0.363	0.037	0.357	8.623	0.396
PBC	family.Ruminococcaceae.id.2050	0.480	0.787	−0.063	0.615	\	\
PBC	genus.Anaerotruncus.id.2054	0.952	0.987	0.057	0.574	1.308	0.987

PBC, primary biliary cholangitis; RSSobs, residual sums of squares of observations; “\”, represents not sufficient snps for analysis.

### 3.3 PSC

Nine bacterial traits were found to be associated with PSC, specifically *order Selenomonadales* (IVW OR 1.72, 95% CI 1.00–2.93, *p* = 0.048), *family Rhodospirillaceae* (IVW OR 1.30, 95% CI 1.01–2.68, *p* = 0.042) and *genus RuminococcaceaeUCG013* (IVW OR 1.63, 95% CI 1.04–2.57, *p* = 0.034) were positively causally associated with PSC.

**Figure 4:**
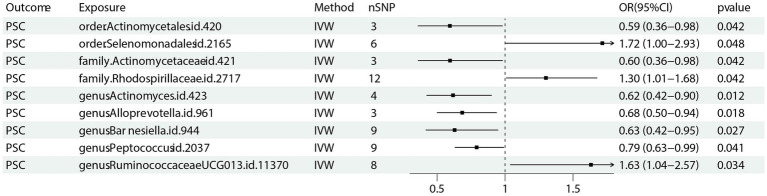
Forest plot of GM taxa associated with PSC identified by IVW method. PSC: primary sclerosing cholangitis; nSNP, number of the single nucleotide polymorphisms; IVW: inverse variance weighted method.

As for *order Actinomycetales* (IVW OR 0.59, 95% CI 0.36–0.98, *p* = 0.042), *family Actinomycetaceae* (IVW OR 1.72, 95% CI 0.36–0.98 *p* = 0.042), *genus Actinomyces* (IVW OR 0.62, 95% CI 0.42–0.90, *p* = 0.012), *genus alloprevotella* (IVW OR 0.68, 95% CI 0.50–0.94, *p* = 0.018), *genus Barnesiella* (IVW OR 0.63, 95% CI 0.42–0.95, *p* = 0.027) as well as *genus Peptococcus* (IVW OR 0.79, 95% CI 0.63–0.99, *p* = 0.041) were found negative association with the risk of PSC (Figure 4) Other results are shown in Supplementary Table 3.

**Figure 5:**
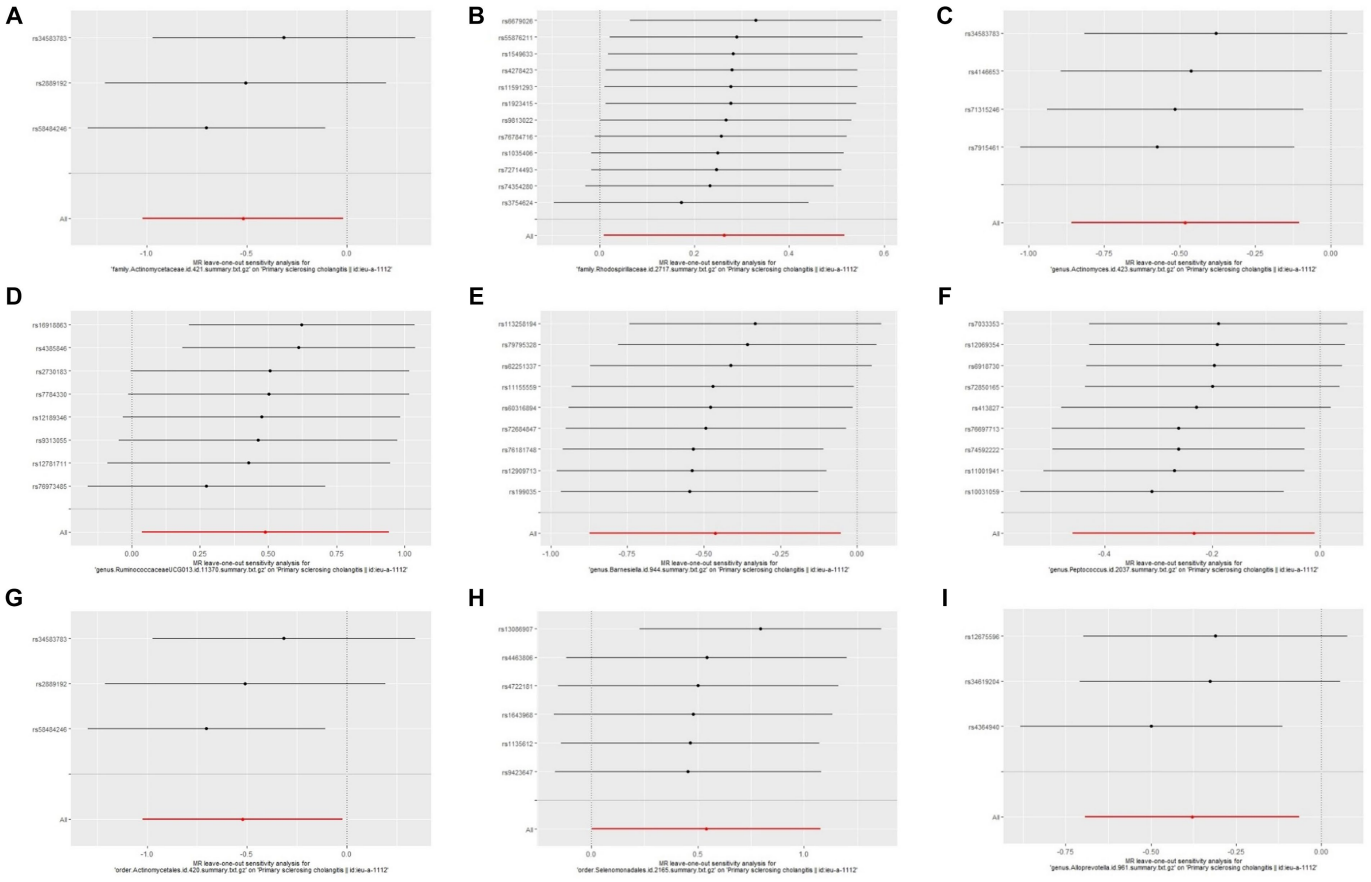
Leave one out analysis of the MR results of GM taxa associated with PSC. **(A)** family.Actinomycetaceae.id.421, **(B)** family.Rhodospirillaceae.id.2717, **(C)** genus.Actinomyces.id.423, **(D)** genus.RuminococcaceaeUCG013.id.11370, **(E)** genus.Barnesiella.id.944, **(F)** genus.Peptococcus.id.2037, **(G)** order.Actinomycetales.id.420, **(H)** order.Selenomonadales.id.2165, **(I)** genus.Alloprevotella.id.961.

Through Cochran’s Q test, we detected no heterogeneity (*p* > 0.05). All *p*-values of the MR-PRESSO test and the MR-egger interpret test were > 0.05, indicating the absence of outliers or pleiotropy (Table 2). We then examined the forest plot and scatter plot (Supplementary Figures 3, 4). Finally, the robustness of our primary findings was validated using the leave-one-out method (Figure 5).

**Table 2:** Sensitivity analysis of gut microbiota on PSC.

Outcome	Exposure	Cochrane’s Q	*p*	Egger_intercept	*p*	RSSobs	*p*
PSC	family.Actinomycetaceae.id.421	1.400	0.497	0.134	0.461	\	\
PSC	family.Rhodospirillaceae.id.2717	8.387	0.678	0.021	0.798	10.170	0.684
PSC	genus.Actinomyces.id.423	1.100	0.777	−0.030	0.772	2.042	0.801
PSC	genus.Alloprevotella.id.961	1.111	0.574	0.253	0.504	\	\
PSC	genus.Barnesiella.id.944	10.122	0.257	0.077	0.264	12.810	0.288
PSC	genus.Peptococcus.id.2037	7.652	0.468	−0.045	0.521	9.874	0.473
PSC	genus.RuminococcaceaeUCG013.id.11370	9.861	0.197	−0.045	0.316	13.929	0.204
PSC	order.Actinomycetales.id.420	1.400	0.497	0.134	0.461	\	\
PSC	order.Selenomonadales.id.2165	5.291	0.381	0.297	0.153	7.349	0.432

PSC, primary sclerosing cholangitis; RSSobs, residual sums of squares of observations; “\”, represents not sufficient snps for analysis.

### 3.4 Reverse Mendelian randomization

A reverse MR analysis was utilized through the IVW method to explore the potential causal association between PBC, PSC, and these specific gut microbial taxa. The data presented in Supplementary Table 4 did not show any significant reverse causal association between PBC, PSC and these specific gut microbial taxa.

## 4 Discussion

In this two-sample Mendelian Randomization study, we found that 7 bacterial taxa were associated with PBC, and 9 bacterial taxa were associated with PSC.

Previous article reported (28) that Order Selenomonadales, Order Bifidobacteriales, Genus Lachnospiraceae_UCG_004, Family Peptostreptococcaceae, and Family Ruminococcaceae were related to PBC, which have been validated in our study. In addition, our results also found that order Bacillales and genus Anaerotruncu were potential risk factor for PBC. These results expand previous research and provide a foundation for further research on PBC, and we also compared the differences between PBC and PSC.

We identified *order Selenomonadales* as a protective factor in both PBC and PSC. *Selenomonadales* are anaerobic bacteria that typically have a curved or bent shape. They contribute to the formation and function of complex gut microbiota. These bacteria can utilize various carbon sources such as glucose, lactose, and cellulose to produce organic acids and gases through fermentation (29).

It was also found that the *family Peptostreptococcaceae* is a protective factor for PBC, while the *genus Peptococcus* has a similar protective effect on PSC. These results indicate that certain gut microbiota might play a common role in the occurrence of PBC and PSC.

The family *Peptostreptococcaceae* belongs to the *phylum Firmicutes* (30). This family includes the *genera Peptostreptococcus*, *Finegoldia*, and *Anaerococcus*. Bacteria in the family *Peptostreptococcaceae* are typically anaerobic organisms and can be found in the digestive tract, skin, and other body surfaces of humans and animals, as well as in soil and water environments.

There are also some bacteria that play different roles in PBC and PSC. For example, the *family Ruminococcaceae* plays a protective role in PBC, while the *genus RuminococcaceaeUCG013* increases the risk of PSC. These results suggest that different bacteria within the same family may also have different effects.

*Family Ruminococcaceae* family are usually anaerobic organisms. These bacteria are able to utilize cellulose and other components of plant cell walls to produce organic acids and gases through fermentation, providing energy and nutrients to the host. These bacteria in the human gut are associated with intestinal health and metabolism (31).

Due to Mendelian randomization analysis using GM GWAS data, different levels of bacteria, such as genus, family, and order, may extract the same SNPs, which results in different levels of bacteria having the same effect. For example, order *Bifidobacteriales* and family *Bifidobacteriaceae* both extract 13 SNPs, and the results indicated the same protective effect on PBC. What’s more, although the number of extracted SNPs differed, *order Actinomycetales*, *family Actinomycetaceae*, and *genus Actinomyces* are all having protective effects on PSC.

*Bifidobacterium* is generally regarded as probiotics and possesses numerous advantages, such as facilitating food digestion, synthesizing vitamins, and augmenting immune system functionality (32). However, our findings provide evidence that the *family Bifidobacteriaceae* may elevate the risk of PBC, which is consistence with previous research which identified *Bifidobacterium* is increased in PBC patients (20).

*Actinomycetales* are widely present in natural environments, including soil, water bodies, and plant surfaces. They can also survive in the bodies of humans and other animals, such as in the oral cavity, intestines, and skin. *Actinomycetales* have the ability to produce some important enzymes and bioactive substances (33). For example, they can produce cellulases, proteases, and acid phosphatases, as well as antioxidants and anti-tumor substances.

We found that specific bacterial features are causally related to the risk of PBC and PSC. The underlying mechanism of the influence of bacteria features on PBC and PSC has been extensively studied. Metabolites, especially short-chain fatty acids (SCFAs), are one of the most crucial factors (34, 35), with butyric acid, propionic acid, and acetic acid being the predominant constituents. A critical function of SCFAs is to act as signaling molecules that regulate the immune system, cellular growth, and metabolic activity of the host (36).

Butyrate, an essential metabolite derived from the GM, contributes to maintaining the integrity gut barrier by supplying energy to colonic epithelial cells. Additionally, it modulates genes associated with the circadian clock, thereby performing its anti-inflammatory function (37). Moreover, it can modulate T cell proliferation and regulate the activation of B cells that produce IL-10 and/or IL-17 (38). These cytokines will aggravate the inflammation of bile duct cells in patients with PBC and PSC and worsen bile stasis. There is also evidence that bacterial-derived peptides induce CD8^+^ T cell clonal expansion (39, 40), which are the main effector cells causing bile duct damage in patients with PBC and PSC (41).

Bile acids also play an important role in the pathogenesis of PBC and PSC. Bile acids not only play a role in digesting food, but also serve as important messengers for liver and intestinal communication.

Firstly, bile acids have a direct bactericidal effect and have an inhibitory effect on the growth of gut microbiota. In addition, bile acids regulate the composition of gut microbiota by regulating farnesoid X receptors. Finally, bile acids can also serve as raw materials for gut microbiota to promote the proliferation of some bacteria (42, 43). Dysbiosis of the GM, characterized by a decrease in microbial diversity and changes in specific bacterial species, may be linked to an elevated risk of developing PSC and PBC (44–46).

Based on our Mendelian randomization study, we have found a reasonable correlation between the gut microbiota taxa, PBC, and PSC. Some probiotics and their metabolites can restore the ecological balance of gut microbiota, repair the intestinal mucosal barrier and regulate systemic immune function. Our research can provide a foundation for further research (47). Future studies are required to better understand the mechanisms behind this prevalent disease and identify potential therapeutic targets.

Our study has some limitations: (a) Since the normally used threshold (*p* < 5 × 10^−8^) did not yield enough IVs, we set a lenient threshold (*p* < 1 × 10^−5^). (b) It’s difficult to determine whether some specific species are related to the outcome since most GM studies using 16S rRNA permit resolution at the genus level. (c) We refrained from conducting multiple corrections in our study. However, it is worth mentioning that the rigorous application of a multiple-testing correction may be excessively conservative and could potentially miss out on partially potential GM taxa that are causally correlated to CLD. Hence, we made the decision not to incorporate multiple correlations. Furthermore, it is important to note that the Bonferroni correction has the possibility of generating false negative results.

## 5 Conclusion

Our findings offer novel evidence that supports the causal influence of particular bacterial abundance on the risk of PBC and PSC. The GM is anticipated to be a promising treatment and prevention target for PBC and PSC.

## Data availability statement

The original contributions presented in the study are included in the article/Supplementary material, further inquiries can be directed to the corresponding authors.

## Author contributions

JY: Data curation, Investigation, Writing – original draft. GM: Investigation, Methodology, Writing – original draft. KW: Software, Writing – original draft. HY: Software, Writing – original draft. SJ: Data curation, Methodology, Writing – original draft. QF: Methodology, Validation, Writing – original draft. XZ: Supervision, Writing – review & editing. GG: Supervision, Visualization, Writing – review & editing. YH: Funding acquisition, Project administration, Writing – review & editing.

## Footnotes

^1^ https://MiBioGenrug.nl
