# Supplementary material for: Causal associations between gut microbiota and Cholestatic liver diseases: a Mendelian randomization study
Source: Front Med (Lausanne). 2024 Jan 24;11:1342119. doi: 10.3389/fmed.2024.1342119 (PMC10847275; doi:10.3389/fmed.2024.1342119)

Supplementary Figure 1 Forest plots of the PBC MR analysis.


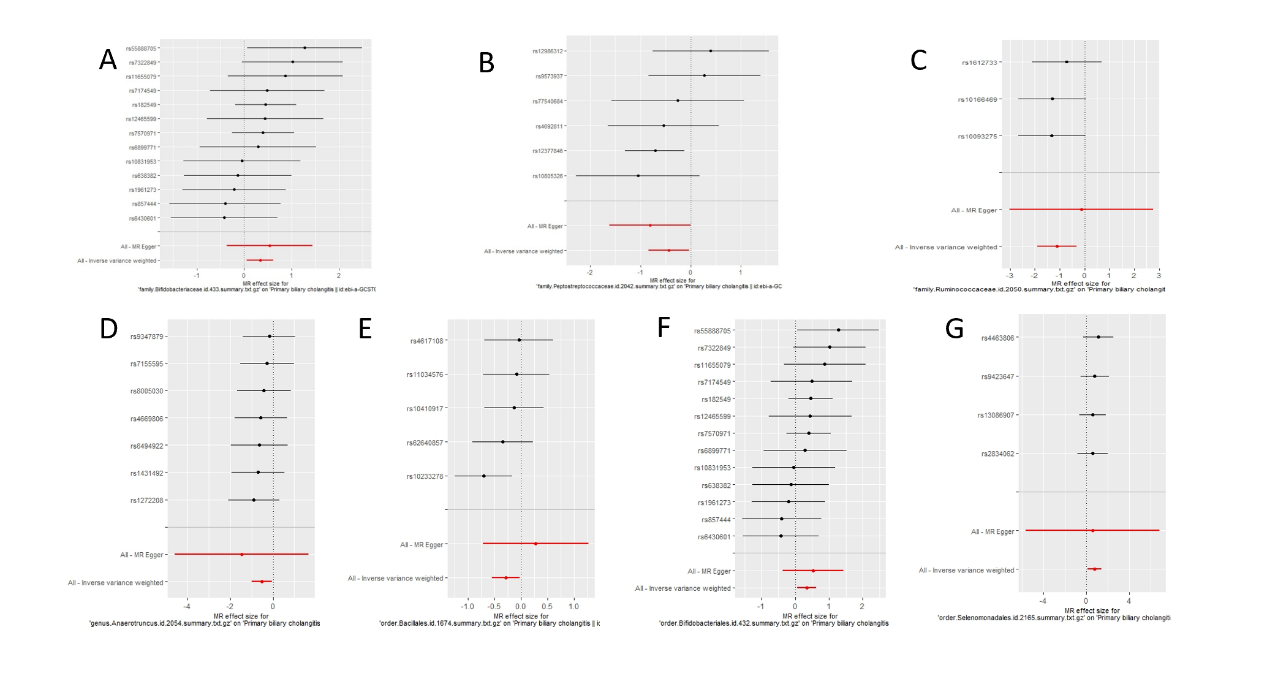


A:family.Bifidobacteriaceae.id.433,B:family.Peptostreptococcaceae.id.2042,C:family.Ruminococcaceae.id.2050 , D:genus.Anaerotruncus.id.2054,E:order.Bacillales.id.1674, F:order.Bifidobacteriales.id.432 ,G: order.Selenomonadales.id.2165

Supplementary Figure 2 Scatter plots of the PBC MR analysis.


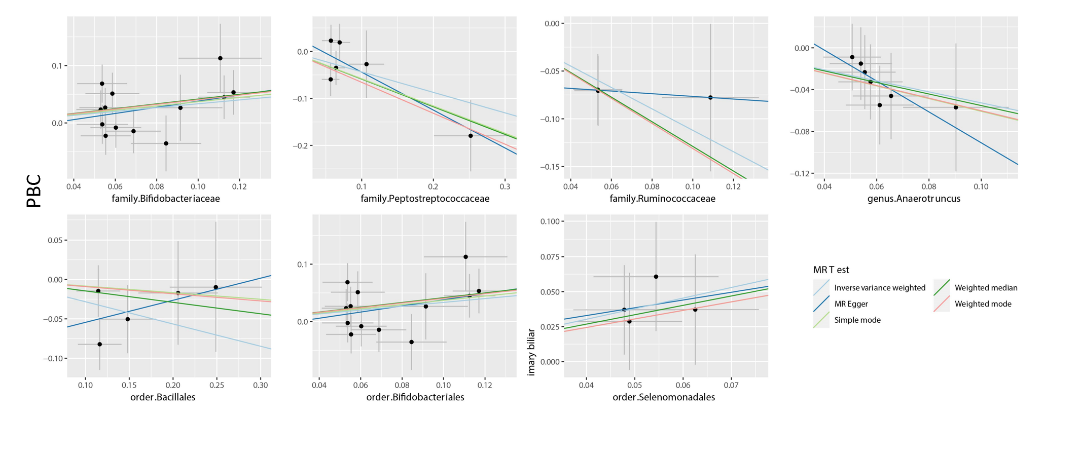


Supplementary Figure 3 Forest plots of the PSC MR analysis.


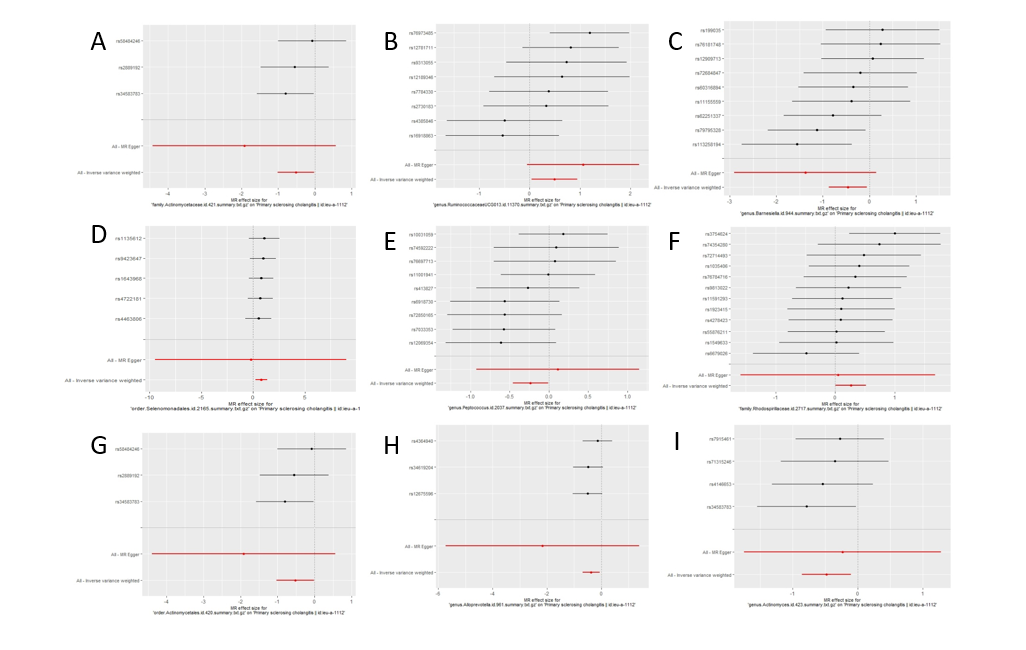


A:family.Actinomycetaceae.id.421,B:genus.RuminococcaceaeUCG013.id.11370,C:genus.Barnesiella.id.944,D:order.Selenomonadales.id.2165,E:genus.Peptococcus.id.2037,F:family.Rhodospirillaceae.id.2717,G:order.Actinomycetales.id.420,H:genus.Alloprevotella.id.961,I: order.Selenomonadales.id.2165

Supplementary Figure 4 Scatter plots of the PSC MR analysis.


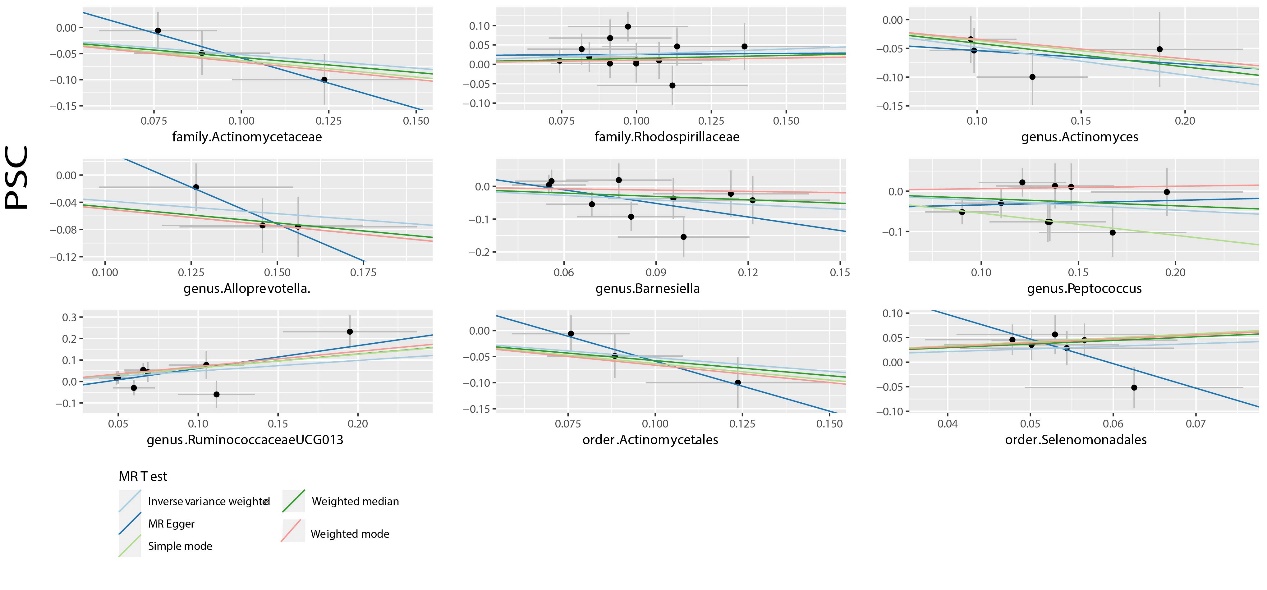

Supplement: Supplementary file 5 [file Data_Sheet_1.docx]
